# Statement of Retraction: Mitogen-activating protein kinase kinase kinase kinase-3, inhibited by Astragaloside IV through H3 lysine 4 monomethylation, promotes the progression of diabetic nephropathy by inducing apoptosis

**DOI:** 10.1080/21655979.2026.2729900

**Published:** 2026-10-01

We, the Editors and Publisher of the journal *Bioengineered*, have retracted the following article from publication.

Fan, Y., Fan, H., Li, P., Liu, Q., Huang, L., & Zhou, Y. (2022). Mitogen-activating protein kinase kinase kinase kinase-3, inhibited by Astragaloside IV through H3 lysine 4 monomethylation, promotes the progression of diabetic nephropathy by inducing apoptosis. *Bioengineered*, 13(5), 11517–11529. https://doi.org/10.1080/21655979.2022.2068822

Since publication, significant concerns have been raised about the integrity of the data and reported results in the article.

When approached for an explanation, the authors did not respond to our queries and so these serious concerns remain unaddressed.

As verifying the validity of published work is core to the integrity of the scholarly record, we are therefore retracting the article. The authors have been informed of this decision.

We have been informed in our decision-making by our policy on publishing ethics and integrity and COPE guidelines. 

The retracted article will remain online to maintain the scholarly record and will be digitally watermarked on each page as ‘Retracted’. 

